# Effect Of Horseradish (Moringa Oleifera) Seeds on Biochemical and Molecular Parameters in Paraquat‐Induced Parkinson Disease‐Like Symptoms in Fruit Flies

**DOI:** 10.1002/alz.088477

**Published:** 2025-01-09

**Authors:** Olayemi Philemon Aro, Adeniyi Stephen Adefegha, Opeyemi Babatunde Ogunsuyi, Ganiyu Oboh

**Affiliations:** ^1^ Universidade Federal do Rio Grande do Sul, Porto Alegre, Rio Grande do Sul Brazil; ^2^ Federal University of Technology, Akure Nigeria; ^3^ Federal University of Technology, Akure, Ondo Nigeria

## Abstract

**Background:**

In recent decades, epidemiological and experimental studies have looked into the role of pesticides, particularly the herbicide paraquat, in the development of Parkinson’s disease. Horseradish tree (*Moringa oleifera)* is an ethnobotanical plant with lots of therapeutic potential, but there is a dearth of information on the bioactive properties of the seed alkaloid extracts.

**Method:**

This study examined the modulatory effects of various concentrations of an alkaloid extract from the seeds of Horseradish Tree (*Moringa oleifera*) on the survival rate of flies exposed to paraquat, as well as certain biochemical and molecular markers related to Parkinson’s disease in the heads of the flies.

**Result:**

The findings showed that the survival rate of the treated flies was significantly (p<0.05) higher than that of paraquat‐induced flies. The catalase and total thiol content of the flies were found to be significantly lower in the paraquat‐induced flies, but this was ameliorated in the treated groups. In addition, when compared to their elevated levels in paraquat‐induced flies, monoamine oxidase and acetylcholinesterase activities in the treated groups were found to be significantly (P<0.05) reduced. In addition, the enhanced reactive oxygen species (ROS) of the paraquat‐induced groups were significantly reduced after being treated with alkaloid extracts. The findings of gene expression demonstrate that catalase and tyrosine hydroxylase (TH) mRNA expression levels were downregulated in the induced group but elevated in the treated groups, with the seed showing a significant upregulation in catalase expression compared to tyrosine hydroxylase (TH) mRNA expression.

**Conclusion:**

According to the findings of this study, alkaloid extracts from *Moringa* seeds ameliorated impaired biochemical indices and, at the same time, upregulated genetic expression in paraquat‐induced flies. The results of this research indicate that extracts from *M. oleifera* seeds have neuroprotective potential, especially as it relates to Parkinson’s disease.

